# Changes in health parameters in older lay volunteers who delivered a lifestyle-based program to frail older people at home

**DOI:** 10.1007/s00508-018-1372-6

**Published:** 2018-08-09

**Authors:** Igor Grabovac, Sandra Haider, Eva Winzer, Ali Kapan, Karin E. Schindler, Christian Lackinger, Thomas E. Dorner

**Affiliations:** 1 Department of Social and Preventive Medicine, Centre for Public Health, Medical University of Vienna, Kinderspitalgasse 15/1, 1090 Vienna, Austria; 2 Department of Internal Medicine III, Division of Endocrinology and Metabolism, Medical University of Vienna, Vienna, Austria; 3 Department for Health Promotion and Prevention, SPORTUNION Austria, Vienna, Austria

**Keywords:** Non-professionals, Physical activity, Nutritional behavior, Frailty, Volunteers

## Abstract

**Objective:**

To measure health effects in lay volunteers who made home visits consisting of social interaction, nutritional and physical exercise interventions to pre-frail and frail older people (trial registration ClinicalTrials.gov, NCT01991639).

**Methods:**

After baseline, participants were followed-up at 12 (V1) and 24 (V2) weeks. A one-repetition maximum (1-RPM) and handgrip were measured with the Concept2**®**DYNO and a dynamometer. The Physical Activity Scale for the Elderly was used to assess physical activity, and Food Frequency Questionnaire and the Mediterranean Diet Adherence Screener for nutrition. Additionally, quality of life (QoL) was measured with the World Health Organization (WHO) quality of life brief questionnaire and anthropometric measurements were performed using bioelectrical impedance analysis.

**Results:**

Handgrip values significantly increased from 32.14 ± 7.94 kg to 33.69 ± 6.72 kg at V1 and 34.36 ± 6.96 kg at V2. The 1‑RPM on the leg press showed a significant increase from 72.47 ± 25.37 kg to 78.12 ± 23.77 kg and 80.85 ± 27.99 kg, respectively. We observed a significant decrease of protein intake from 0.38 ± 0.26 g/kgBW/day to 0.32 ± 0.19 g/kgBW/day and 0.26 ± 0.16 g/kgBW/day, respectively. There were no changes in physical activity, QoL and anthropometric measurements.

**Conclusion:**

The findings indicate that projects involving aging healthy volunteers may have additional limited health benefits.

## Introduction

The beneficial effect of physical exercise and nutritional optimization on the health of older people has been previously described [1, 2]; however, these interventions were mainly conducted by healthcare professionals and consequently the health effect on those who deliver health promotion has seldom been examined. Health interventions that are based on peer support from non-professionals have been shown to have an effect mostly in studies concerning diabetes mellitus and drug adherence with the outcomes usually focusing on those receiving the support [3, 4]. Awareness of the peer support system’s influence on salutogenic effects is gaining more importance as the focus in healthcare shifts from treatment-based to health promotion [5]. To further underline this need for strengthening of support and social relationships, the World Health Organization (WHO) has outlined strengthening of social relationships as a valuable health promotion strategy, while the enrichment of supportive resources through mutual aid has been endorsed by the Ottawa Charter [6, 7].

Studies have linked volunteering to numerous protective factors on mental and physical health. A prospective study showed that when controlling for different factors, people who scored high on volunteering scales had 44% overall lower mortality than non-volunteers [8]. Some of the positive effects of volunteering on psychological well-being have been identified in studies that linked volunteering to greater self-esteem, greater life satisfaction and a reduction in symptoms of depression [9]; however, volunteering was also shown to have an effect on physical health not only by increasing psychological well-being, but by additionally improving the level of physical activity, as many volunteering jobs require moderately intense physical activity. Also, volunteering provides with ideas and alternative sites for physical activity. There is also a growing number of research projects showing an overall increase in physical activity in older volunteers than in those who do not volunteer [10, 11]. Overall volunteers reported positive effects on self-rated health status, mortality and adoption of healthier life styles, e.g. nutrition outcomes, QoL, depression, psychological distress, and self-esteem [12]. As shown, there is a lot of literature on psychological and self-reported health benefits of volunteering, while information on measurable health benefits is broadly missing.

Considering the reported positive influence of volunteering, the same was expected from participants of a program shown to improve health, well-being, physical fitness and nutrition of prefrail and frail individuals [13–15]. Frailty, a geriatric syndrome characterized by an increased vulnerability to external stressors is associated with an accelerated decrease of physiological reserves [16, 17]. There are various methods to assess frailty, one of them is the SHARE-FI [18], with which one can classify people as robust, prefrail (a pre-stage of frailty) and frail [19]. As sarcopenia, malnutrition and chronic inflammation contribute to the frailty syndrome, a combination of strength training and nutritional intervention (focused on appropriate protein intake) have been shown to be effective [1, 2]. In such a randomized controlled trial examining the effects of nutritional and physical exercise intervention in pre-frail and frail community-dwelling older and very old persons, healthy non-professional older volunteers (buddies) were recruited to visit the prefrail and frail subjects twice a week and perform a life style-based intervention, consisting of physical training, nutritional optimization, and social support [20]. The effects on the health status of the prefrail and frail subjects have been reported previously [13–15]. In this study, our aim was to assess health parameters, inducing physical fitness, body composition and nutritional parameter of the volunteers who visited the prefrail and frail individuals.

## Methods

### Participants

The volunteers, so-called buddies, were recruited with the help of a local volunteer organization providing care to old people (“Wiener Hilfswerk”). Participants answered advertising campaigns published in newspapers as well as informative articles in brochures. Furthermore, the program was presented in several television programs. Inclusion criteria were: older than 50 years of age, readiness to participate in both the control and the intervention group and willingness to participate for the full duration of the program intervention (6 months). Before the buddies started with the home visits, they received 4 training sessions over 4 weeks (each about 3 h long). These training sessions aimed to provide the buddies with the knowledge and skills to conduct home visits. After the training session, buddies were matched with a prefrail or frail partner, and the couple randomly assigned in an intervention or a control group. With prefrail or frail individuals in the intervention group, the buddies had to conduct six strength exercises and had to cover nutritional topics. With prefrail or frail individuals in the control group they were to conduct home visits with social support for the first 12 weeks. Afterwards they performed the same physical training and nutritional intervention. The training sessions covered topics of aging, malnutrition, importance of healthy nutrition and physical exercise, basics of physical training, as well as motivational techniques. Importance of appropriate execution of strength exercises until muscular exhaustion was carried out in practical sessions, where they also received instructions on how to teach the techniques. These were held by professionals (medical doctor, nutritionist, nutrition scientists, sports scientist, physiotherapist, and psychologist) and written materials designed for the study were given out to the participating buddies. All buddies were also trained concerning healthy nutrition. In this context buddies got information about topics such as proper hydration, protein and energy intake. Additionally, themes such as fruit and vegetables intake and “extras” (e.g., food with high fat, high salt and high sugar content) were addressed. As the control group, started with the nutritional and physical exercises 12 weeks after the intervention group, an additional refresher session was given to the buddies allocated to the control group.

During the home visits, buddies were asked to perform the same 6 strength exercises as prefrail individuals in 2 sets with 15 repetitions until exhaustion. Those were mini squats in front of a chair, chest presses against elastic resistance, an exercise for the abdominal muscles (performed sitting on a chair), hip extensions in standing position, reverse butterfly and shoulder presses against elastic resistance. If the described exercise was too easy, buddies were also provided with an alternative for each exercise, aiming to train the same muscle group. Apart from exercising during the home visits, buddies were asked to perform the same exercises once a week at home (without the frail persons), irrespective of the group assignment. Additionally, as described, they were trained concerning healthy eating in later adulthood and were asked to implement these nutritional guidelines in their everyday life.

### Measurements

Measurements in the buddies were conducted 3 times during the study duration: at baseline, after the first 12 weeks (visit 1) and after 24 weeks (visit 2). These were done by physical measurements of muscle strength and body impedance analysis as well as a number of questionnaires. Handgrip strength was assessed in kilograms (kg) using a hydraulic hand dynamometer from Jamar (Lafayette Instrument, Lafayette, Louisiana, USA), while the participants were sitting [21]. The strength of each hand was alternately tested three times, with a break of 1 min in between. The highest of all six values was taken for the calculation. The one repetition maximum (1-RPM) and muscular endurance were measured by using the “Concept2 DYNO” (Concept 2, Morrisville, Vermont, USA) dynamometer. One repetition maximum and muscular endurance were measured for three different exercises with their respective muscle groups. These included the seated squat (for lower limb muscles), bench press (pectoral muscles), and bench pull (upper back muscles). Using the “Concept2 DYNO” which uses a flywheel to create resistance ranging from 0 to 500 kg, dynamic measurements of the 1‑RPM and muscular endurance could be undertaken. A total of three submaximal warm-up lifts were done before the participants were instructed to do one repetition with maximum force possible (which is equal to their 1‑RPM). For the muscular endurance 15 consecutive repetitions with maximum voluntary effort were performed with the mean weight as the indicator of muscular endurance [22].

Anthropometry: body height was measured with a tape, and body weight with a Marsden MS-4203 (Marsden Group, Rotherham, UK) calibrated scale allowing body mass index (BMI) to be calculated. Abdominal girth was measured by a tape at a level 2.5 cm vertically higher than the umbilicus in expiration [23].

Body composition was measured by bioelectrical impedance analysis (BIA) in a standardized procedure involving the participants lying on their back with electrodes put on the dominant hand and foot. By using an alternating current body resistance and reactance are assessed. The BIA 2000-S device from Data Input (Darmstadt, Germany) was used in our study [24]. For our study; phase angle (PA), lean body mass and fat mass index were variables chosen. Although the pathological effects of the PA have not been clarified it has been interpreted as a sign of cell death or increased permeability of the cell membrane and is also been found to be a good prognostic marker of disease progression and survival. It was also reported that the PA increases with improvements in the clinical health status [25, 26]. The fat mass index was found to be a good indicator in assessment of body adiposity and is calculated as the body fat mass value of the BIA divided by the height squared [27]. Finally, the lean body mass in kg was calculated by using the formula “total body water/0.73” [28].

Physical Activity Scale for the Elderly (PASE): is a 12-question self-report questionnaire that measures physical activity in individuals aged 65 years and over in items that are organized regarding occupational, leisure and household activities during the last 7‑day period. Items are differently scored where leisure and strengthening activities are scored as never, seldom (1–2 days per week), sometimes (3–4 days per week) and often (5–7 days per week). Duration is then scored as less than 1 h, 1–2 h, 2–4 h or more than 4 h. Household and work-related items are scored as “yes” and “no”, where in work-related activities paid and unpaid work are again scored on the basis of duration. Overall score ranges from 0 to 400 with higher scores indicating more physical activity [29].

Mediterranean Diet Adherence Screener (MEDIAS): is a 14-item screener questionnaire consisting of questions about food amount intake and adherence to a Mediterranean diet. The answers are scored from 0–1 making the possible final score as anywhere between 0 and 14 points [30].

Protein intake was assessed by using the protein-containing food groups of the European Prospective Investigation into Cancer and Nutrition Study Food-Frequency Questionnaire (EPIC FFQ; [31]). The participants indicated the number of times a given food item was consumed, where one portion size corresponds for example to one palm of the hand, which was presented by an image in the questionnaire. A total of 39 food items which corresponds to 5 food groups as “meat and meat products and eggs” (9 items), “fish” (8 items), “milk and milk products” (7 items), “legumes and nuts” (6 items) and “cereals and bread” (9 items) were assessed. Each food item of the FFQ represents an individual food (for example salmon) and one question about the frequency and type of commonly consumed dietary supplements was added to the FFQ. Furthermore, the frequencies of food items were calculated as the daily intake. The German Nutrient Database “Bundeslebensmittelschlüssel” (BLS, version 3.01) was used to obtain the protein intake of one portion size (g per day). The daily protein intake was further calculated in g protein per kg bodyweight per day (g/kg BW/day). Moreover, the dietary protein intake was categorized in plant and animal-based protein intake (g/kg BW/day). Additionally, serum albumin concentration (analyzed by a licenced biomedical laboratory) was used to assess the nutritional status.

World Health Organization Quality of Life Scale Brief Version (WHO-QOL-Bref): a shorter version developed from the original WHO-QOL-100 measures quality of life in 4 domains: physical, psychological, social and environmental and a fifth overall QoL and general health domain were also calculated. Questions are on a Likert type scale (ranging from 1 as “completely disagree” to 5 “completely agree”) where the participants show their agreement or disagreement with a given statement. Final score is a sum of points, reverse scoring is applied where appropriate [32].

Social Support Questionnaire (*Fragebogen zur sozialen Unterstützung*; F‑Sozu): for this study, the short 14-item form was used, which was derived from the original 54-item version, and is recommended for research with limited time, but with validity and reliability remaining at a high level. The questionnaire consists of 14 statements, where on a Likert type scale the participants indicate their agreement or disagreement ranging from 1 “fully applies” to 5 “does not apply” [33]. Based on the median split and the percentiles the scores are divided into categories based on the level of social support needed.

### Statistical analysis

As buddies of the intervention and also the control group were asked to implement the physical training exercises and the nutritional topics in their everyday life from the very beginning of the study, they were considered as one group and were analyzed as such. Descriptive statistics were done for each variable and presented as means, standard deviation in metric variables and frequencies in categorical variables. The relationship between handgrip strength and leg press muscle strength was investigated using Pearson’s product-moment correlation coefficient. Changes over time for relevant variables were calculated by the paired samples t‑tests and the Wilcoxson-Mann-Whitney test for non-parametric distribution in metric variables. Differences in categorical variables were calculated with the χ^2^-test. For all calculations, a statistical probability of *p* < 0.05 was considered significant. Statistical software package SPSS 20.0 (SPSS, Chicago, IL, USA) was used.

## Results

A total of 84 buddies were recruited with 74 (88.1%) being female and with a mean age of 60.06 ± 6.93 years. Of these 38 (54.3%) finished secondary school followed by 15 (21.4%) participants who reported having a primary and 13 (18.6%) a tertiary education level. At baseline the BMI was measured with a mean of 25.64 ± 5.39 kg/m^2^ and abdominal girth with 93.31 ± 14.85 cm.

A significant increase in muscle strength was observed with the leg press between baseline measurements and visit 1. Results of changes in hand grip strength over time show a significant change between baseline and visit 1 as well as baseline and visit 2, with a steady increase in mean strength values as seen in Table 1. There was a strong positive correlation between handgrip strength and 1 RPM of the leg press muscle strength; at baseline r = 0.550; *p* < 0.001, at visit 1 r = 0.643; *p* < 0.001 and visit 2 r = 0.637; *p* < 0.001, with higher values of handgrip indicating higher values for muscle strength in the lower extremities.

**Table 1:** Changes over time in outcome parameters

Variable	Baseline (B)	Visit 1 (3 Months)	Visit 2 (6 Months)	*P *(B-visit 1)^a^	*P *(B-visit 2)^a^
Muscle strength (kg)					
Bench press	31.41 ± 11.70	31.16 ± 8.27	30.05 ± 7.36	0.839	0.250
Bench pull	35.95 ± 13.05	36.02 ± 9.24	34.64 ± 7.87	0.943	0.189
Leg press	72.47 ± 25.37	78.12 ± 23.77	80.85 ± 27.99	0.001*	0.223
Hand Grip	32.14 ± 7.94	33.69 ± 6.72	34.36 ± 6.96	0.003*	0.037
Muscle endurance (kg)					
Bench press	22.46 ± 6.45	22.45 ± 6.45	22.73 ± 6.24	0.324	0.796
Bench pull	26.45 ± 7.58	25.68 ± 8.05	25.44 ± 6.51	0.175	0.763
Leg press	56.05 ± 21.08	58.50 ± 18.65	58.63 ± 17.75	0.618	0.401
Body composition					
Phase angle (grade)	5.41 ± 0.62	5.36 ± 0.59	5.41 ± 0.66	0.186	0.374
Lean body mass (kg/m^2^)	50.03 ± 7.57	49.40 ± 7.83	49.87 ± 8.14	0.673	0.682
Fat mass index (kg/m^2^)	7.69 ± 4.04	7.79 ± 3.82	7.55 ± 3.85	0.021*	0.970
PASE (h/week)					
Total score	229.44 ± 118.31	224.83 ± 117.22	236.75 ± 176.14	0.357	0.074
Strength training	1.31 ± 2.48	1.39 ± 1.64	0.67 ± 0.98	0.664	0.199
MEDIAS categories (%)				0.092	0.982
No adherence	13.4	24.3	11.3		
Low adherence	68.7	47.1	52.8		
High adherence	17.9	11.4	11.3		
Missing	0	17.1	22.6		
Protein intake (g/kgBW/day)	0.38 ± 0.26	0.32 ± 0.19	0.26 ± 0.16	0.041*	0.004*
Plant-based protein intake (g/kgBW/day)	0.20 ± 0.13	0.18 ± 0.11	0.16 ± 0.11	0.014*	0.008*
Animal-based protein intake (g/kgBW/day)	0.18 ± 0.22	0.14 ± 0.15	0.10 ± 0.10	0.200	0.062
Serum albumin concentration (g/l)	44.98 ± 2.55	44.44 ± 1.98	44.37 ± 2.17	0.091	0.973
WHO-QOL-Bref score					
Overall health and quality of life	60.59 ± 12.04	60.52 ± 13.82	64.05 ± 13.01	0.909	0.254
Physical domain	85.44 ± 10.77	83.89 ± 13.18	86.26 ± 12.81	0.444	0.534
Psychological domain	75.98 ± 10.62	75.86 ± 13.22	77.50 ± 13.56	0.880	0.867
Social support domain	55.89 ± 10.07	54.02 ± 11.28	56.79 ± 10.22	0.094	0.664
Environmental domain	84.84 ± 9.39	83.65 ± 10.91	84.11 ± 10.06	0.026*	0.723
F-Sozu categories (%)				0.098	0.706
Very low	82.1	75.7	67.9		
Low	2.4	7.1	7.5		
Normal	2.4	0	0		
Missing	13.1	17.1	24.5		

Data are presented as mean (standard deviation) for metric variables and percentage for categorical variables

*PASE* Physical Activity Scale for the Elderly, *WHO-QOL-Bref* World Health Organizations Quality of Life Brief, *MEDIAS* Mediterranean Diet Adherence Screener, *F-Sozu* “Fragebogen zur sozialen Unterstützung” (social support questionnaire), *B* baseline, *h/week* hours per week, *kg* kilogram, *g/kgBW/day* grams per kilogram bodyweight per day, *g/l* grams per litre, *kg/m*^*2*^ kilograms per square metre

^a^Paired samples t‑test, Mann-Whitney-U test or χ^2^-test as appropriate

* Significant results

As presented in Table 1, phase angle and lean body mass values were consistent during all three measurement points with no statistically significant changes found across time. The only change found in body composition parameters was the fat mass index where a significant increase was observed between baseline and the first follow up visit at 12 weeks; however, no differences were observed between baseline and final follow up measurement at week 24.

In Table 1 the PASE score is presented with two values: the total PASE score indicating overall physical activity and the PASE score of the strength training variables. Although not found to be significant, both scores showed a decline between baseline and visit 2, with the strength training score at almost 50% its baseline values.

No significant changes between visits were observed regarding the MEDIAS score (Table 1). Protein intake presented as grams per kilo of bodyweight per day shows a significant decrease between visits. There was also an observed significant decrease in protein intake from plant and a remarkable, but not statistically significant decrease in animal-based protein sources (Table 1). Serum albumin concentration found at 44.98 ± 2.55 g/l at baseline also showed a slight but not significant decrease between baseline and visit 1 and visit 2 as shown in Table 1.

In quality of life scores, we only observed a significant decrease in the environmental domain of the WHO-QOL-Bref between baseline and visit 1. No other significant changes were observed. We also found no significant changes in social support as measured by the F‑SOZU (Table 1).

## Discussion

In this study, we found significant gain in upper and lower extremity strength, and an unexpected change in dietary pattern in a sample of older volunteers who performed a comprehensive life style-based health promotion project with older prefrail and frail and subjects. To our surprise, in most of the recorded parameters no statistically significant changes could be observed. Nonetheless, the presented study reveals several interesting findings.

Regarding muscle strength, the baseline results were in line with reference values for 1‑RPM in an Austrian female population aged 60–69 years with 33.0 ± 6.4 kg for bench press, 37.3 ± 7.5 kg for bench pull and 67.4 ± 15.5 kg for leg press [34]. The only significant change in this study was an increase in muscle strength in the thighs and handgrip. Especially handgrip is of great importance, because handgrip strength has been found to be a reliable parameter related to bone density, cardiovascular and cancer mortality, frailty, etc. [35–37]; however, muscle strength results for bench press and pull were not found to differ over time. Additionally, there was no significant change in muscle endurance. This lack of effect can be partly explained when we look at the total PASE score that measures physical activity and even more so the PASE score for strength training. While not significant over time, there was a noticeable change in time spent on strength training where the hours per week spent for strength training at visit 2 were almost half of the baseline results, meaning that the participants did not perform the exercises as instructed. Some of the participants reported to the research team being overwhelmed by participation in the study and said that they stopped doing the exercise regimens that they did before being included in the study. Additionally, some of the participants concentrated on providing the correct intervention to the frail persons and lacked the focus in performing their own exercises with the necessary intensity.

In the study participants, there was no change in phase angle between study visits. In one reference study the phase angle in Caucasian women aged 60–69 years old was found to be around 5.97° ± 0.83° [38]. The mean baseline phase angle was therefore in line with this study. The fat mass index (FMI) has been used by researchers as a better way of determining obesity in comparison to the widely used BMI. The FMI was found to be larger in women in comparison to men and was also found to increase with age. A study by Schutz et al. in 2002 used dual-energy X‑ray absorptiometry (DXA), which was found comparable to BIA results [39]. With these reference values our study results fall between the 50th and the 75th percentile [39]. Also, of all the BIA results the FMI was found to be significantly different between baseline and visit 1 where an increase can be seen. Additionally, no differences in lean body mass (LBM) were found between visits. As no effect on the amount of physical activity, measured by the PASE and no effect on the majority of strength parameter was found, the stable LBM values are explainable.

Protein intake showed a decrease during the duration of the study. At visit 1 it was lower than 50% of the recommended intake of 0.8 g/kg body weight/day [40], and was even lower in visit 2. Similarly, the serum albumin decreased (not significantly) from baseline to visit 1 and visit 2 and remained consistently within the reference values [41]. The reasons for these results can only be hypothesized. Some misconceptions about nutrition did arise during conversation with the participants. As most of the participants considered themselves to be health-conscious (based on the question “would you say that at the present you lead a healthy life style” 71.4% responded affirmatively) they based their diet on vegetables and fruit with little meat. The very intense schooling about healthy nutrition, together with the desire to eat healthy could have led the participants to foster a lower intake of meat and other protein sources. This can also be seen in the self-reporting of the sources of protein, where we observed a significant decrease in consumption of plant-based protein sources, and a remarkable (but not significant) reduction in animal-based protein sources. Another reason may be that the timing of the first measurement overlapped with the training sessions. It is also possible that at the beginning of the study the buddies put an emphasis on protein-rich diet, which diminished over the study period or the buddies merely indicated consuming more protein rich nutrients at the beginning, because the awareness of protein importance was higher due to the training sessions. Finally, it is conceivable that during the project, the buddies became convinced that they lead healthy life styles, that their particular interest in health nutrition decreased and they changed their diet patterns to include less protein but more carbohydrates and fat, which may also explain why their FMI increased significantly during the first study period. The relationship between muscle mass and strength with protein intake is known and has been shown in postmenopausal women where protein intake of ≥1.2 g/kg body weight/day was connected to higher muscle strength [42]. Therefore, this low protein intake might be a contributing factor to the overall lack of improvement in some muscle strength and in all muscle endurance values.

No significant changes were found in WHO-QOL-Bref (except the decrease in the environmental domain between baseline and visit 1), MEDIAS or F‑Sozu questionnaires. From the baseline results it is visible that the participants were men and women of good general health who were health-conscious and lived active lives. Therefore, this type of intervention during 6 months would, presumably, not lead to significant changes in the measured outcomes.

Lastly, study limitations should be addressed. The fact that the study participants did self-administrated questionnaires (PASE, FFQ, MEDIAS, WHO-QOL-Bref) might lead to some reporting bias meaning that some of the results might be even lower. The underrepresentation of men in our study sample is also a factor that may prohibit the generalizability of this study. Additionally, in the present study we did not distinguish between the intervention and control group; however, we recommended all volunteers, irrespective of the group, to conduct the exercises from the very beginning of the study.

## Conclusion

Working voluntarily as buddies for a home-based life style program for older prefrail and frail people led to significant improvements in only few health parameters for the volunteers, whereas most measured health parameters did not change. Similar research needs to focus more on providing better support for the volunteers and giving the right information and optimal amount of training.
